# 300. Clinical Manifestations and Risk Factors for Treatment Failure in Native Vertebral Osteomyelitis: A 25-Year Multicenter Mayo Clinic Experience

**DOI:** 10.1093/ofid/ofaf695.102

**Published:** 2026-01-11

**Authors:** Takahiro Matsuo, Fabio Borgonovo, Brian Lahr, Francesco Petri, Rita Igwilo-Alaneme, Sergio L Alvarez Mulett, Amin Alavi, Aaron J Tande, Elie F Berbari

**Affiliations:** Mayo Clinic, Rochester, MN; Mayo Clinic, Rochester, MN; Mayo Clinic, Rochester, MN; Mayo Clinic, Rochester, Minnesota, Rochester, Minnesota; Mayo Clinic, Rochester, MN; Mayo Clinic, Rochester, MN; Ahvaz Jundishapur University of Medical Sciences, Ahvaz, Alborz, Iran; Mayo Clinic, Rochester, MN; Mayo Clinic, Rochester, MN

## Abstract

**Background:**

Native vertebral osteomyelitis (NVO) is a life-threatening spinal infection with considerable clinical burden. Although increasing literature has described its features and associated treatment failure, large-scale cohorts with long-term follow-up remain limited. Herein, we evaluated a large cohort of patients with NVO to characterize the clinical manifestations, management, and long-term outcomes, and to further explore factors associated with treatment failure.

**Table 1.**
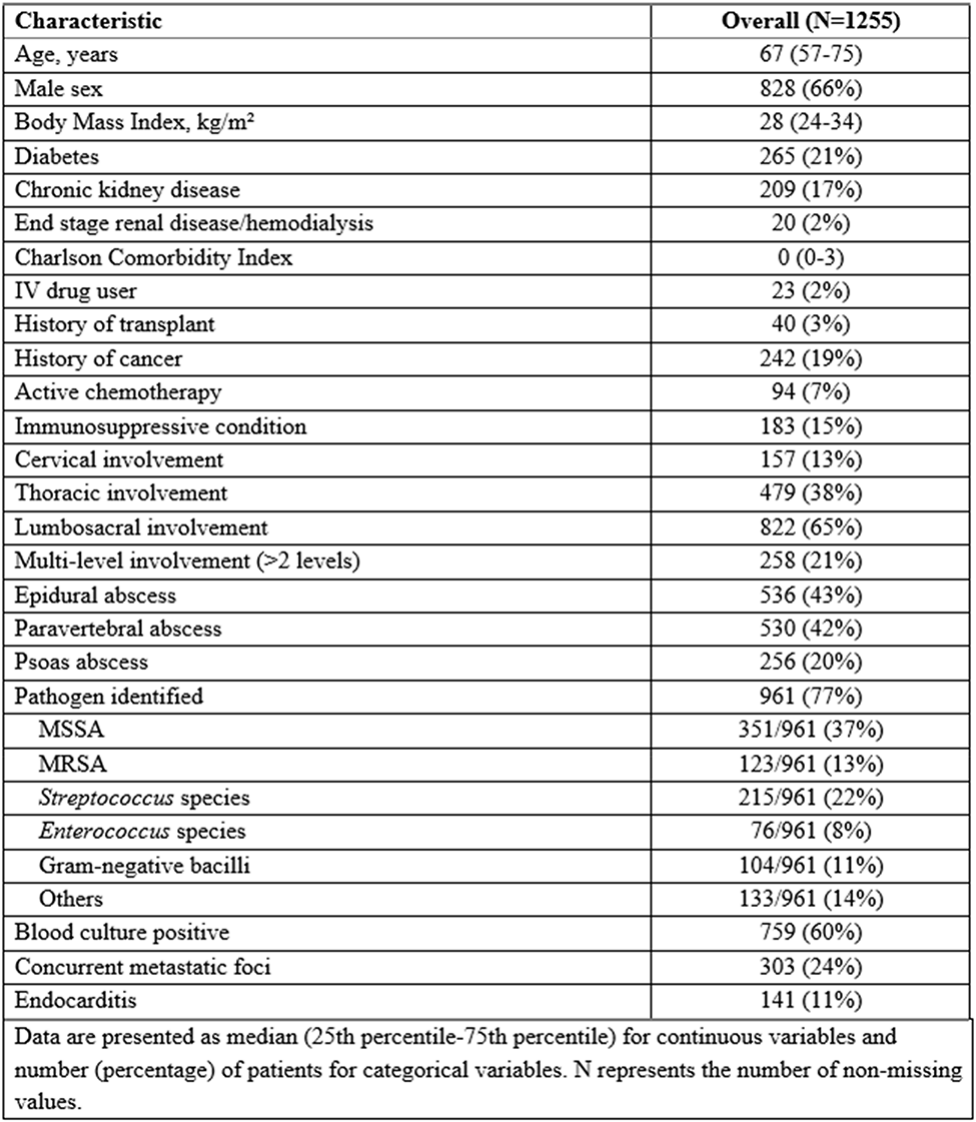
Baseline characteristics of patients with native vertebral osteomyelitis

**Table 2.**
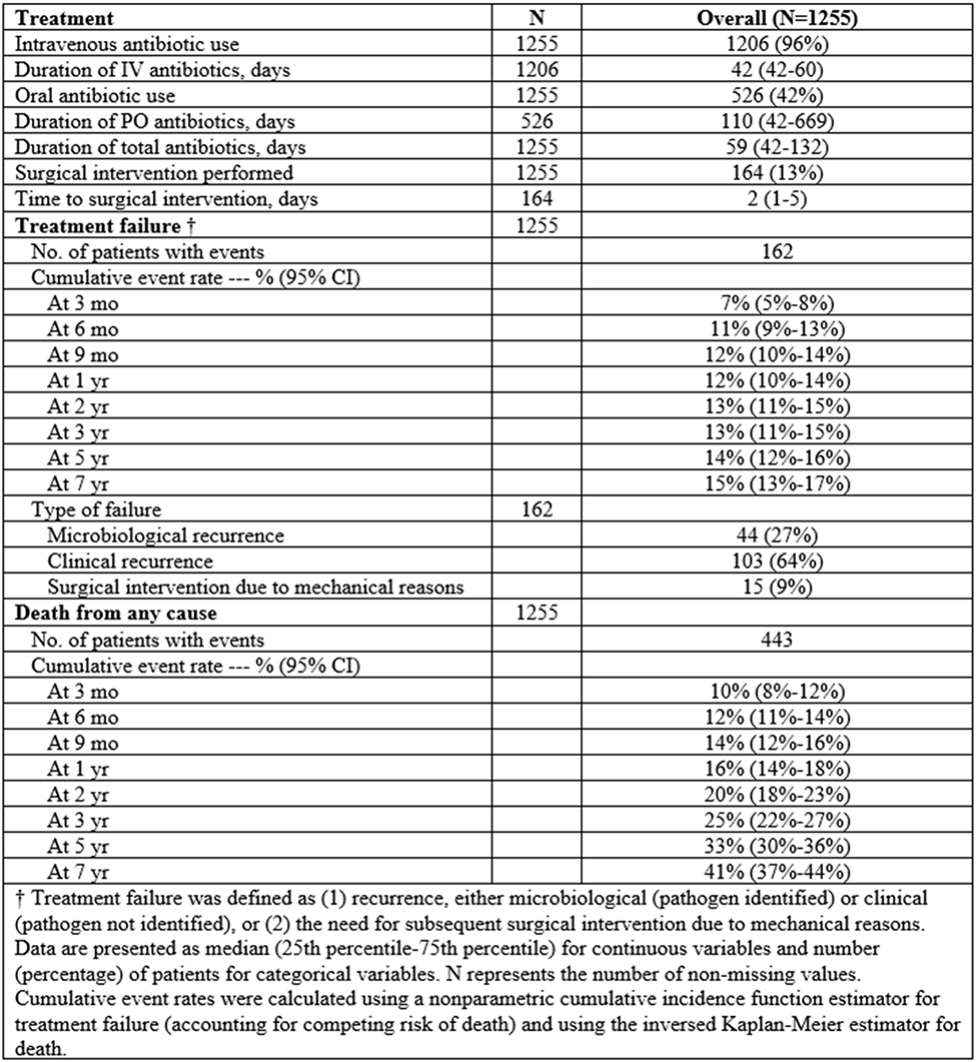
Treatment, failure rates, and mortality in patients with native vertebral osteomyelitis

**Methods:**

We conducted a retrospective multicenter cohort study of adults (≥18 years) with NVO across Mayo Clinic sites between 1999 and 2024. Data on demographics, clinical manifestations, antimicrobial therapy, and surgical intervention were collected. Cumulative incidence of treatment failure was estimated as a function of follow-up accounting for the competing risk of death. Independent predictors of treatment failure were evaluated in a multivariable Cox regression model with time-dependent covariates.

**Table 3.**
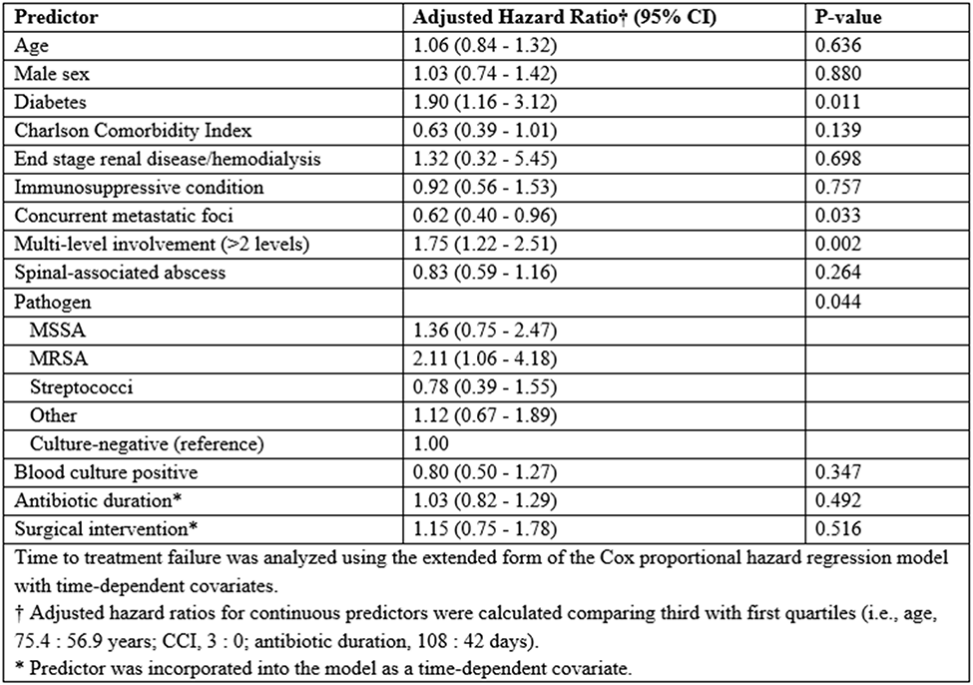
Multivariable predictors of treatment failure in patients with native vertebral osteomyelitis

**Figure 1.**
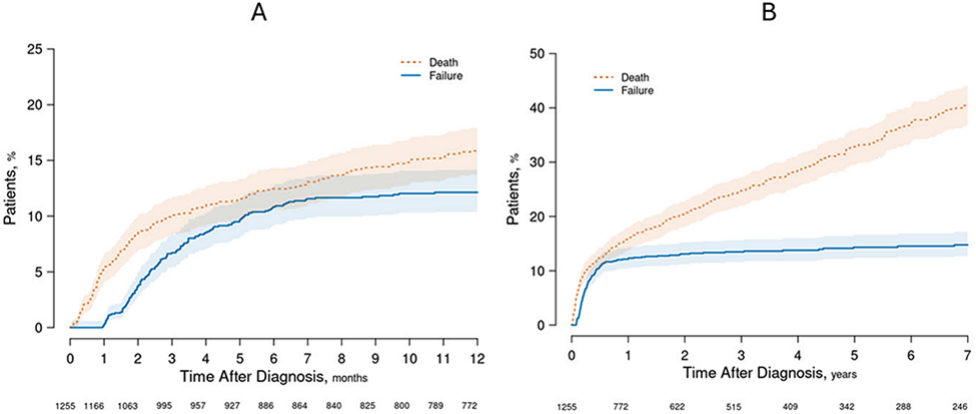
Cumulative incidence of death and treatment failure following diagnosis of native vertebral osteomyelitis (A) Short-term (12-month) cumulative incidence of death and treatment failure (magnified view of the first year from panel B). (B) Long-term (up to 7 years) cumulative incidence of death (dotted orange line) and treatment failure (solid blue line).

**Results:**

Among 1,255 patients included (median age 67 years; 66% male), lumbosacral involvement was most common (65%), with multilevel vertebral involvement ( >2 levels) in 21%. Epidural abscesses were present in 43%, paravertebral abscesses in 42%, and psoas abscesses in 20%. Pathogens were identified in 77%, most commonly *S. aureus* (50%; MSSA 37%, MRSA 13%), followed by *Streptococcus* species (22%), and Gram-negative bacilli (11%) (Table 1). The median total antibiotic duration was 59 days (IQR, 42–132). Surgical intervention was performed in 13% after a median of 2 days (IQR, 1-5). Treatment failure occurred in 162 patients during a median follow-up of 2.8 years (6-month and 5-year cumulative incidence were 11% and 14%, respectively), with the median time to failure of 3.1 months. Cumulative mortality was 12% at 6 months and 33% at 5 years (Table 2, Figure 1). Diabetes (HR 1.90, 95% CI 1.16–3.12), multilevel vertebral involvement (HR 1.75, 95% CI 1.22–2.51), and MRSA infection (HR 2.11, 95% CI 1.06–4.18) were independently associated with increased risk of treatment failure (Table 3).

**Conclusion:**

This 25-year study highlights the persistent burden of treatment failure in NVO and associated risk factors, underscoring the importance of early recognition of high-risk features to optimize outcomes.

**Disclosures:**

All Authors: No reported disclosures

